# Evaluation of MC4R [RS17782313, RS17700633], AGRP [RS3412352] and POMC [RS1042571] polymorphisms with obesity in Northern India

**DOI:** 10.1186/1755-8166-7-S1-P104

**Published:** 2014-01-21

**Authors:** Apurva Srivastava, Balraj Mittal, Jai Prakash, Neena Srivastava

**Affiliations:** 1 Department of Physiology, King George’s Medical University, Lucknow, (U.P), India; 2 Department of Pediatrics, King George’s Medical University, Lucknow, (U.P), India; 3 Department of Medical Genetics, SGPGIMS, Lucknow, (U.P), India

## Background

Genetic variants of the melanocortin-4 receptor gene (MC4R), agouti related protein (AGRP) and proopiomelanocortin (POMC) are reported to be associated with obesity. Therefore, we examined MC4R rs17782313, MC4R rs17700633, AGRP rs3412352 and POMC rs1042571 for association with obesity in North Indian individuals.

## Material and methods

The variants were investigated for association in 300 individuals with BMI≥30kg/m^2^ and 300 healthy non-obese individuals with BMI<30kg/m^2^. The genotyping were analyzed by Taqman probes. The statistical analysis was performed by means of the software SPSS, ver.19 and P≤ 0.05 was considered statistically significant.

## Results

The genotypes of MC4R rs17782313 and POMC rs1042571 were significantly associated with obesity (BMI≥30kg/m^2^) (p=0.02; OR=1.7 and p=0.01; OR=1.6 respectively). However, MC4R rs17700633 (p=0.001; OR=0.55) was associated with low risk. AGRP rs3412352 (p=0.93; OR= 0.96) showed no association with obesity (BMI≥30kg/m^2^) in North Indian individuals.

## Conclusions

The study provides the first report of association of MC4R rs17782313 and POMC rs1042571 that these may have an effect on obesity BMI≥30kg/m^2^ but MC4R rs17700633 and AGRP rs34123523 may not have any influence on obesity BMI≥30kg/m^2^ in North Indian individuals.

